# Research on the Effect of Desert Sand on Pore Structure of Fiber Reinforced Mortar Based on X-CT Technology

**DOI:** 10.3390/ma14195572

**Published:** 2021-09-25

**Authors:** Fangying Shi, Tianyu Li, Weikang Wang, Ruidan Liu, Xiaoyan Liu, Huiwen Tian, Nazhen Liu

**Affiliations:** 1 College of the Environment, Hohai University, Nanjing 210098, China; 2014010202@hhu.edu.cn; 2 College of Mechanics and Materials, Hohai University, Nanjing 210098, China; 201308030004@hhu.edu.cn (R.L.); liuxiaoyan@hhu.edu.cn (X.L.); 3 School of Water Resources and Hydropower Engineering, Wuhan University, Wuhan 430070, China; wkwang@whu.edu.cn; 4 Key Laboratory of Marine Environmental Corrosion and Bio-Fouling, Institute of Oceanology, Chinese Academy of Sciences, Qingdao 266071, China; tianhuiwen@qdio.ac.cn

**Keywords:** desert sand, X-CT, MIP, pore structure, porosity, mechanical property

## Abstract

Concrete is a multi-phase, porous system. The pore structure has an important influence on the properties of the concrete. In this paper, a kind of fiber reinforced mortar was prepared with desert sand and its pore structure was studied. The MIP technique was used to investigate the pore structure characteristics between 1 nm and 500 μm (in diameter). Meanwhile, the μX-CT technique was used to study the pore structure characteristics above 200 μm. It was found that the total porosity tends to decrease first and then increase as the dosage of desert sand increased. The porosity decreased gradually from the upper to bottom area inside the sample, and the diameter of the air voids near the upper area became larger. After curing for 28 days, the compressive strength of fiber reinforced mortar reached the maximum when the content of desert sand was 50%. In conclusion, the appropriate amount of desert sand can reduce the porosity of the fiber reinforced mortar to some extent and the number of large size air voids can be significantly reduced, which improves the pore structure and the mechanical properties of the fiber reinforced mortar.

## 1. Introduction

The annual use of construction sand in China has increased more than 20 times in the past 30 years and it is expected to grow by more than 20% a year [1]. Increasing demand for construction sand has led to a shortage of river sand, and overmining of river sand has negatively affected the ecological environment [2]. The contradiction between supply and demand of construction sand is increasingly prominent with the rapid increase of engineering quantity and the relevant management regulations of sand and stone collection by the state [3]. If the construction sand can be partially or completely replaced by desert sand, the environmental pressure of desertification in the desert could be reduced while protecting the original ecological environment [4]. Nowadays, scholars have made great progress in the application research of desert sand in concrete. Zhang et al. [5] found that the sand of the Tengger and Mause desert could be used for plaster mortar engineering when the glue sand ratio was greater than 1:2. Jin et al. [6] studied the mechanical properties of concrete using Maousu sand as fine aggregate, arguing that desert sand can act as a fine aggregate for concrete in general civil engineering. Researchers [7,8,9] proved that sand in the desert can be used to make concrete mixtures without harmful effects. In addition, Arroudj et al. [10] used desert sand for ultra-high-performance concrete and found that when desert sand was combined with the addition of amorphous morphology (blast slag or silica fume), it can compact the microstructure of concrete and improve the strength. According to the single factor test of Yang et al. [11], the impact of desert sand replacement rate on the compressive strength of high-strength concrete showed a trend of first increasing and then decreasing.

Concrete is a multi-phase, porous system. The inner pore structure has the important influence on the property of the concrete [12,13,14,15], such as the property of the concrete, deformation behavior, water absorption, freezing resistance, permeability, and durability, etc. [16]. The effect of the pore structure on concrete has long been regarded as an important factor affecting the macroscopic behavior of concrete. With the development of scientific research activities [17,18,19], it has been found that the increase in porosity reduces the strength of concrete, and the degree of this effect depends largely on the size, shape, and spatial distribution of pores. As the study of pore structure deepened, more and more theories and methods were introduced into the research of pore structure. The common pore methods include optical method, mercury intrusion porosimetry (MIP), isothermal adsorption method and X-ray small angle scattering [16]. Existing porosity-strength relationships (e.g., Ryshkewithch, Schiller, Balshin, and Hasselman models) have been proposed. Chen et al. [20] used Zheng’s multiporosity material model to evaluate the relationship between cement mortar porosity and cement mortar strength, and illustrated the effect of porosity on concrete strength. The mercury intrusion porosimetry (MIP) is currently the most used and effective method to study the pore cascade. BU et al. [21] developed a statistical model that can link compressive strength to associated pore structure features by MIP tests. The results showed that porosity structure is also an important reason affecting the compressive strength of concrete except porosity. Based on the MIP test and the fractal model, Jin et al. [22] obtained the relationship between the fractal dimension of the pore surface and the pore characteristic parameters of the cement mortar. Liu et al. [23] used MIP to test the pore structure of non-dispersible underwater concrete and revealed the relationship between pore structure and permeability of concrete, indicating that the addition of slag powder can ameliorate the pore size distribution of non-dispersed underwater concrete, reduce the porosity, and make the concrete structure more compact, which is beneficial to improving the permeability resistance of concrete at the macro level. However, the diameter range of MIP measurable pores is commonly between 5 and 750 μm. Pores greater than this range cannot be measured [16].

X-ray Computed Tomography (X-CT) is a kind of nondestructive testing technology which obtains the internal structure image of an object using computer reconstruction and an X-ray energy source. Currently, X-CT has been used to investigate the microstructure of cement-based materials. According to the resolution, it can be divided into micron CT (resolution of μm/pixel) and nano CT (resolution of nm/ pixel level). In recent years, X-CT has been gradually applied to research in the field of building materials, such as cement hydration, characterization of pore structure, transition zone of mortar interface, geometric distribution of fibers in concrete, sulfate erosion, steel corrosion, carbonization, etc., [24,25,26,27,28,29,30,31]. Jun et al. [32] used X-CT to research the relationships between pore structure fractal dimension and pore structure parameters and introduced the calculation method of fractal dimension according to MATLAB. Wang et al. [33] studied the spatial distribution of steel fibers and bubbles in UHPC samples by X-CT technology. Kang [34] studied the pore structure of UHPC in the range of 3–10 mm (in diameter) by X-CT and MIP, and found that each method provided different total porosity and pore size distribution due to different measured pore sizes.

The pore structure has a great influence on the mechanical properties of concrete. This paper aims to study the influence of desert sand on the pore structure of fiber reinforced mortar and evaluate the influence of pore structure on the mechanical properties of desert sand fiber reinforced mortar. In this paper, mercury intrusion porosimetry (MIP) and micron grade X-ray Computed Tomography (μX-CT) were used to study the pore structure of fiber reinforced mortar with different desert sand replacement rate. The MIP technique was used to study the pore structure characteristics in the sample within 1 nm–500 μm size (in diameter), μX-CT technique is used to study the pore structure characteristics with size above 200 μm. The influence of pore structure on the mechanical properties of desert sand concrete is discussed by analyzing the mechanical properties of samples.

## 2. Materials and Methods

### 2.1. Raw Materials

The cement in this study was ordinary Portland cement with a grade of 42.5 (P.O 42.5 cement, OPC). The basic physical properties of cement were shown in Table 1. The steel fiber length was 13 mm, the fiber diameter was 0.2 mm, the fiber aspect ratio was 65, and the tensile strength was more than 2850 MPa. The desert sand used in this study was Maowusu desert sand with the fineness modulus of 0.254 and the mud content of 0.25% without cleaning. The fineness modulus of river sand was 2.2–2.5, and the mud content was 1.5%. The water used in this study was deionized water. Raw material composition and specimen treatment before test of concrete with different mix ratio are shown in Table 2.

We mixed the cement, desert sand (river sand), and steel fiber well with a blender. Then, we added deionized water, mechanically stirred it for 8–10 min evenly, and obtained the fiber reinforced mortar. Maintenance was performed after formwork and vibration. The specimens were 40 mm × 40 mm × 40 mm cubes and 50 mm cylinders with standard curing. Standard curing specifically means that after the test block is formed for 24 h, the mold is dismantled and maintained in the standard curing room for 27 days. The room temperature in the standard curing room should be maintained at 23 ± 2 °C and the humidity should not be less than 95%. The compressive strength of 3 d, 7 d, and 28 d was tested. After curing, MIP and μX-CT tests were performed on five groups of samples.

### 2.2. Test Methods

#### 2.2.1. Compressive Strength

In this paper, mechanical properties are expressed in the form of compressive strength. Compressive strengths of fiber reinforced mortar with desert sand (0, 25, 50, 75, and 100 wt.% replacement) at 3, 7, and 28 days were investigated systematically. Com-pressive strengths were determined in accordance with ASTM C109 and tested at 3, 7, and 28 days. At each age, the final compressive strength data was the average of three parallel samples.

#### 2.2.2. Mercury Intrusion Porosimetry (MIP)

In order to understand the influence of different amounts of desert sand on the pore structure of cement hydration process, samples at 28 days were characterized using mercury intrusion porosimetry (MIP). The MIP test was performed according to ISO 15901-1:2016. For this test, small cube samples were cut into approximately 5 mm × 5 mm × 5 mm from the original cylindrical samples. The sample of this size almost fills the penetrometer. It therefore requires less mercury to fill it. The employment of large single samplse promotes the accuracy of the entire analytical process by reducing the compressibility effects of mercury and increasing the accuracy of density measurements. In order to remove moisture, the samples were dried at 50 °C in a vacuum oven for 48 h. MIP tests were performed by using a AutoPore IV 9510 mercury intrusion porosimeter (Micromeritics Instrument Corporation, Norcross, GA, USA).

#### 2.2.3. μX-CT

In this study, the Siemens Somatom Sensation 40 CT machine (Siemens, Germany) was adopted to obtain the composition spatial distribution of ingredients and meso-structure information of five specimens. This X-ray CT system is based on cone-beam scanning technology, which consists of a 240 kV/320 W microfocus X-ray source and a radiation detector with a nominal resolution of less than 2 μm. This microfocused X-ray source has a resolution of 1 μm and a minimum distance of 4.5 mm between the focus and the sample. In the experiment, 190 kV lamp voltage and 0.45 mA current value were used. After the CT system was ready, the cylindrical sample was secured to a table on a low-density poly-cylindrical base. In order to receive the X-ray radiation beam evenly in the acquisition system, each specimen was moved up and down automatically during the 1 h scan.

##### Porosity Calculation Based on Two-Dimensional CT Images

Before calculating the plane porosity of two-dimensional images, the obtained CT images should be binarized first, and then Image-Pro Plus software (6.0, Media Cybernetics, Rockville, MD, USA) was used to make statistics on the number of pores and particle pixels of the binarized images to calculate the plane porosity. After image processing, the total pore area can be obtained. The plane porosity can be calculated by Equation (1).

(1) $$\rho =\frac{S_{\mathrm{pore}}}{S}\times 100\%$$

where the molecules represent the total area of the pores, and S represents the area of the concrete sample sections in the CT image.

**Figure 1:**
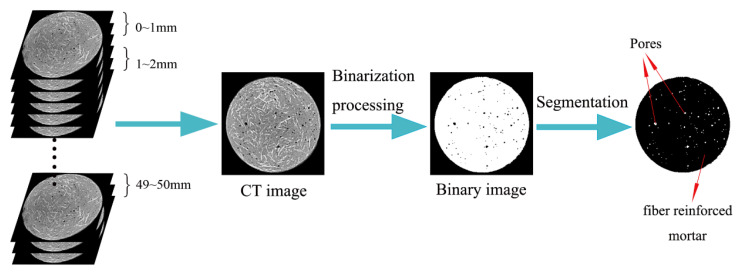
The main process of analyzing the CT image.

On the other hand, 1 sample was divided into 50 groups at 1 mm intervals from the upper end of the sample. The porosity of each group was obtained by calculating the average of plane porosity of CT images in each depth range to study the pore structure characteristics in different positions inside concrete. Figure 1 shows the main process of the CT image analysis.

##### Modeling and Analysis of Pore Structure Based on Avizo Software

**Figure 2:**
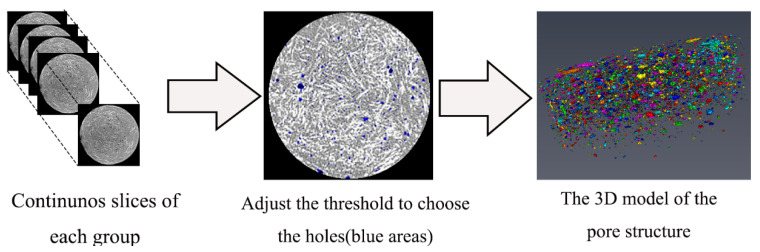
Schematic diagram of the pore structure reconstruction modeling.

In order to analyze the three-dimensional pore structure of fiber reinforced mortar with different desert sand replacement rate, the 3D models of the pore structure of each samples were developed based on continuous slices obtained by μX-CT, and this process was completed by Avizo software (FEI SAS, Villebon sur Yvette, France). Firstly, the threshold was adjusted and selected, and the pores were separated from the solid structure to obtain the 3D reconstruction models of pore structure of each samples. The pore structure statistics were also obtained through the numerical analysis function brought by the software, through which the pore structure of the sample can be analyzed quantitatively. Figure 2 is the schematic diagram of the pore structure reconstruction modeling.

## 3. Results and Discussion

### 3.1. Compressive Strength

**Figure 3:**
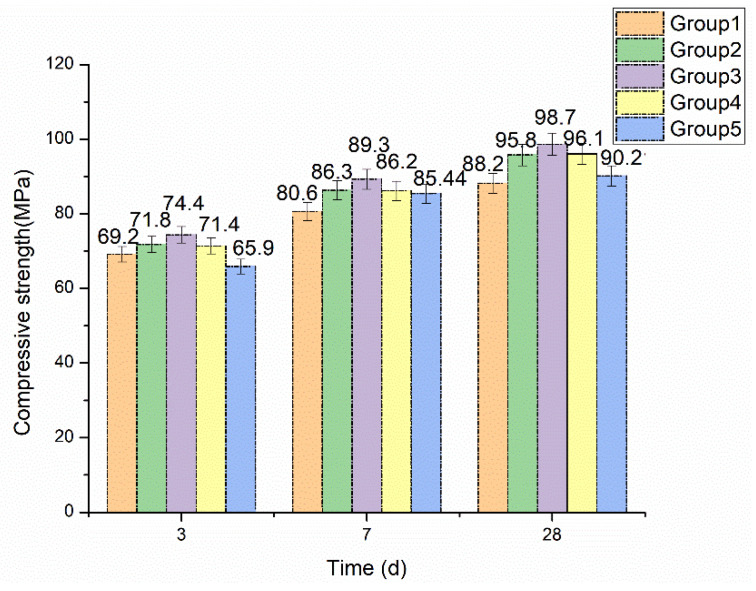
The compressive strength change with the content of desert sand.

The mechanical properties of the samples were expressed in terms of compressive strength. The compressive strength of 3 d, 7 d, and 28 d were shown in Figure 3. It can be seen that the compressive strength of each sample increases with the increase of curing age. At the same curing time, the compressive strength increases first and then decreases with the increase of desert sand replacement rate. The use of desert sand significantly improved the compressive strength of fiber reinforced mortar, and the strength of all samples were higher than that of pure river sand samples after curing for 7 days. After curing for 28 days, relative to river sand fiber reinforced mortar, the increment of compressive strength of fiber reinforced mortar with 25%, 50%, 75%, and 100% desert sand replacement rate was 8.61%, 11.90%, 8.96%, and 2.28%, respectively. These results indicated that the mechanical properties of fiber reinforced mortar with different amounts of desert sand replacement rate are better than pure river sand fiber reinforced mortar, and the improvement effect increased first and then weakened. Fiber reinforced mortar with 50% desert sand replacement rate had the highest compressive strength.

### 3.2. Analysis of MIP

Table 3 shows the pore structure characteristics of five samples based on the MIP test. As can be seen from Table 3, the replacement of river sand by desert sand has the significant impact on the porosity and cumulative pore volume of fiber reinforced mortar. The increment in mortar porosity of 25%, 50%, 75%, and 100% desert sand incorporation was −7.67%, −9.18%, 7.41%, and 17.65%, respectively. With the increase of desert sand replacement rate, the porosity and cumulative pore volume of concrete tended to decrease first and then increase overall. Due to the difference in particle size between desert sand and river sand, the fine aggregate gradation of the two materials is formed after mixing to achieve the effect of dense accumulation, and this effect is best when the ratio of the two materials is 1:1. When the desert sand replacement rate is 50%, due to the small diameter of desert sand particles, they are fully and randomly filled into the pores of the concrete internal structure, thus greatly improving the dense degree, the porosity and cumulative pore volume to achieve the minimum value. The minimum porosity was 12.7888%, and the minimum cumulative pore volume was 0.0662 mL/g. On the other hand, with the increase of sand replacement rate, the mean pore diameter also decreased first and then increased. The minimum mean pore diameter was 40,598 nm.

**Figure 4:**
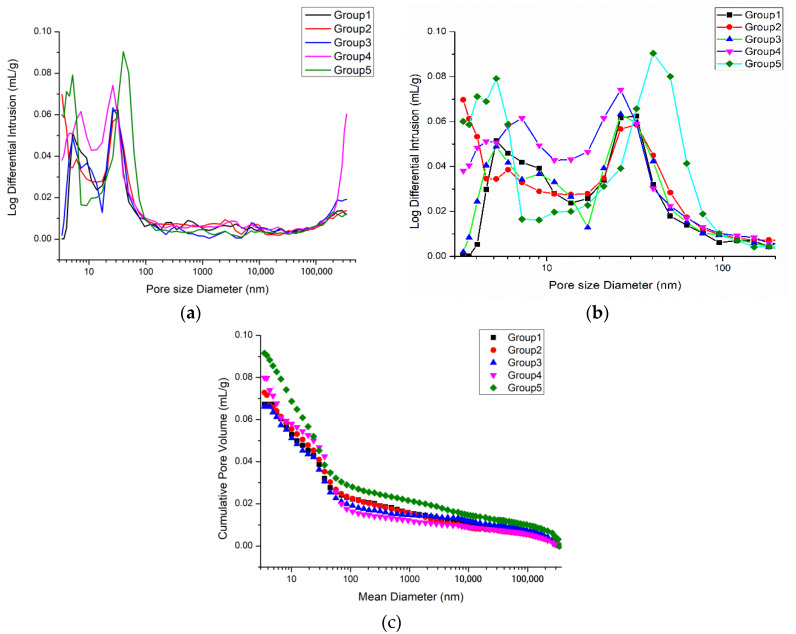
MIP test results. (**a**) The complete MIP test results of pore size distribution of different fiber reinforced mortar. (**b**) The partial MIP test results of pore size distribution of different fiber reinforced mortar. (**c**) Cumulative pore volume distribution curve.

Figure 4 shows the pore size distribution integral curve of five groups of fiber rein-forced mortar. It can be seen that the curves of Groups 4 and 5 were relatively higher. Figure 4a shows the five groups of fiber reinforced mortar comparison, which shows that the pore size changes of the five samples were mainly distributed in the range of 1–100 nm and above 200 μm. We separately analyzed pores with size of 1–100 nm, as shown in Figure 4b. The pore structure of Groups 1–3 was essentially the same in this range, while the curves of Groups 4 and 5 were higher, indicating that the cumulative pore volume in this pore size range is larger. Group 4 peaked around 8 nm and 25 nm, and Group 5 peaked around 4 nm and 40 nm. According to the theory of pore classification proposed by Wu [35,36] in 1973 based on the influence of different pore size on the performance of concrete, the pores below 20 nm are called harmless pores, the pores with pore size between 20 nm and 50 nm are called less harmful pores, the pores with pore size between 50 nm and 200 nm are called harmful pores, and the pores above 200 nm are called more harmful pores. The peaks in Group 5 corresponding to the pore size were greater than the peaks corresponding to Group 4, with a larger pore size being more harmful to the concrete performance. Moreover, the cumulative volume of Group 5 at the peak was also larger, which will affect the performance of fiber reinforced mortar to some extent. When the desert sand replacement rate was 0–50%, the pore structure in the range of 1–100 nm of fiber reinforced mortar was optimized. When the desert sand replacement rate was 50–100%, the pore structure in the range of 1–100 nm of fiber reinforced mortar turned worse.

**Figure 5:**
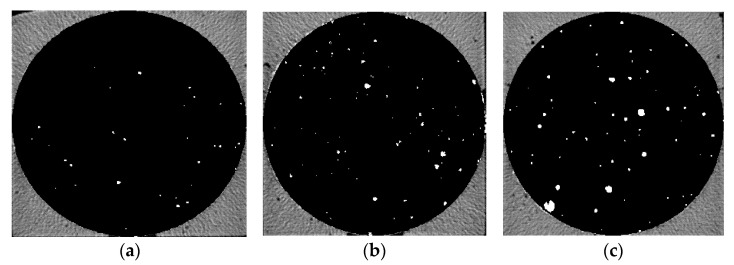
Pore distribution in CT image of Group 1. (**a**) the bottom area; (**b**) the middle area; (**c**) the upper area.

Figure 5 is the cumulative pore volume distribution curve of five samples. The reduced cumulative pore volume of the concrete indicates that the pore content in the concrete pore structure decreases, and the smaller the pore size distribution indicates that the pores of the concrete tend to be harmless pores. It can be seen that when the desert sand replacing rate was 100%, the cumulative pore volume of concrete harmless pores, less harmful pores, harmful pores and more harmful pores increased significantly, indicating that the pore structure of Group 5 was poorer, which will have a certain impact on the mechanical properties of the fiber reinforced mortar. The pore structures in the size range of 1–500 μm of five samples were analyzed using an MIP test. The pore structure changes of the five samples were mainly distributed in the range of 1–100 nm, and there were few or almost no pores in the range of 100–200 μm. When the desert sand replacement rate was 0–50%, the pore structure in the range of 1–200 μm of fiber reinforced mortar was optimized. When the desert sand replacement rate was 50–100%, the pore structure in the range of 1–200 μm of fiber reinforced mortar deteriorated.

### 3.3. Analysis of μX-CT

#### 3.3.1. Pore Characteristic Analysis Based on Two-Dimensional CT Images

**Figure 6:**
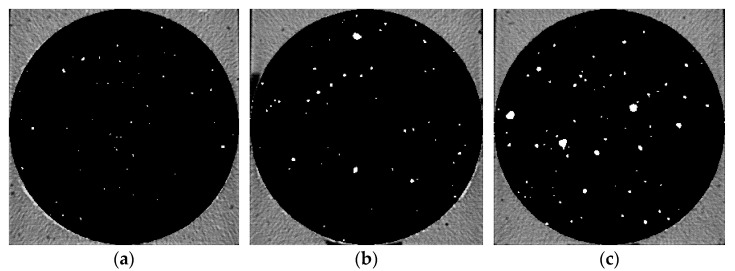
Pore distribution in CT image of Group 2. (**a**) The bottom area; (**b**) the middle area; (**c**) the upper area.

**Figure 7:**
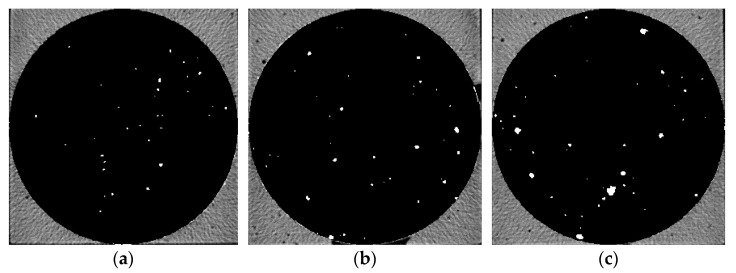
Pore distribution in CT image of Group 3. (**a**) The bottom area; (**b**) the middle area; (**c**) the upper area.

**Figure 8:**
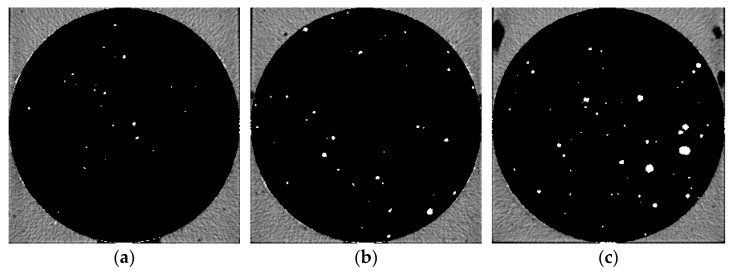
Pore distribution in CT image of Group 4. (**a**) The bottom area; (**b**) the middle area; (**c**) the upper area.

**Figure 9:**
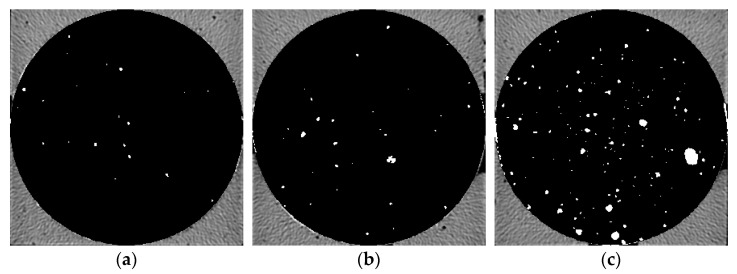
Pore distribution in CT image of Group 5. (**a**) The bottom area; (**b**) the middle area; (**c**) the upper area.

**Figure 10:**
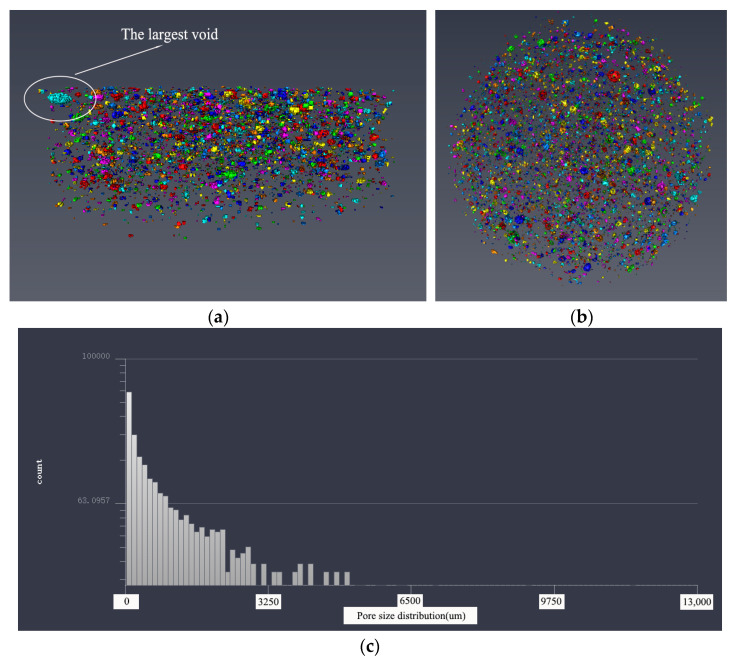
The 3D model of the pore structure of Group 1. (**a**) Side view; (**b**) vertical view; (**c**) pore distribution statistics.

Five groups of samples after 28 d curing time were pretreated and then μX-CT test was performed to analyze the pore structure. Figure 6, Figure 7, Figure 8, Figure 9 and Figure 10 show the pore structure characteristics at the bottom area, middle area and upper area of the five samples (white areas indicate pores). As can be observed in the figure, the air voids in the specimen were mainly concentrated in the middle and upper part of the specimen due to the vibration during the preparation. The closer to the upper area, the larger the size and the number of the air voids. When the replacement rate of desert sand was 0–50%, the number of air voids decreased obviously with the increase of desert sand content, and the number of large voids in the middle and upper areas also decreased obviously. When the desert sand replacement rate was 50–100%, the number of air voids began to increase again, and the number of larger voids also increased. Although the pore structure in the specimen can be analyzed to some extent from the two-dimensional CT image, the most intuitive visual effect and qualitative and quantitative analysis results cannot be fully obtained.

In order to obtain quantitative pore structure characteristic information, porosity calculation was carried out according to Section 2.2.3 based on two-dimensional CT images. The porosity for the different depth ranges of the five samples are shown in Table 4. According to the calculation results of pore structure characteristics, the porosity of the fiber reinforced mortar with different desert sand replacing rate was between 0.73% and 4.00%. When the desert sand replacement rate was 0%, the maximum porosity at different depths was 3.88%, the minimum porosity was 0.74%, and the maximum porosity was 5.24 times of the minimum porosity. When the desert sand replacement rate was 25%, the maximum porosity at different depths was 3.37%, the minimum porosity was 0.73%, and the maximum porosity is 4.62 times of the minimum porosity. When the desert sand replacement rate was 50%, the maximum porosity at different depths was 3.21%, the minimum porosity was 0.69%, and the maximum porosity was 4.65 times of the minimum porosity. When the desert sand replacement rate was 75%, the maximum porosity at different depths was 3.33%, the minimum porosity was 0.73%, and the maximum porosity was 4.56 times of the minimum porosity. When the desert sand replacement rate was 100%, the maximum porosity at different depths was 4.00%, the minimum porosity was 0.75%, and the maximum porosity was 5.33 times of the minimum porosity.

With the increase of desert sand replacement rate, the maximum and minimum porosity of fiber reinforced mortar at different depths and the gap between them first decreased and then increased. Controlling the amount of desert sand replacement rate invariant, the porosity tended to decrease with depth. It was obvious that porosity in every sample: the upper area > the middle area > the bottom area. With the increase of desert sand replacement rate, the total porosity decreased first and then increased. The decrease of porosity indicates that the pore structure of the sample is optimized. The porosity of Group 3 was the lowest, which was 30% lower than that of river sand mortar, indicating that the addition of desert sand can improve the pore structure of fiber reinforced mortar to a certain extent.

#### 3.3.2. Pore Structure Analysis Based on 3D Modeling

**Figure 11:**
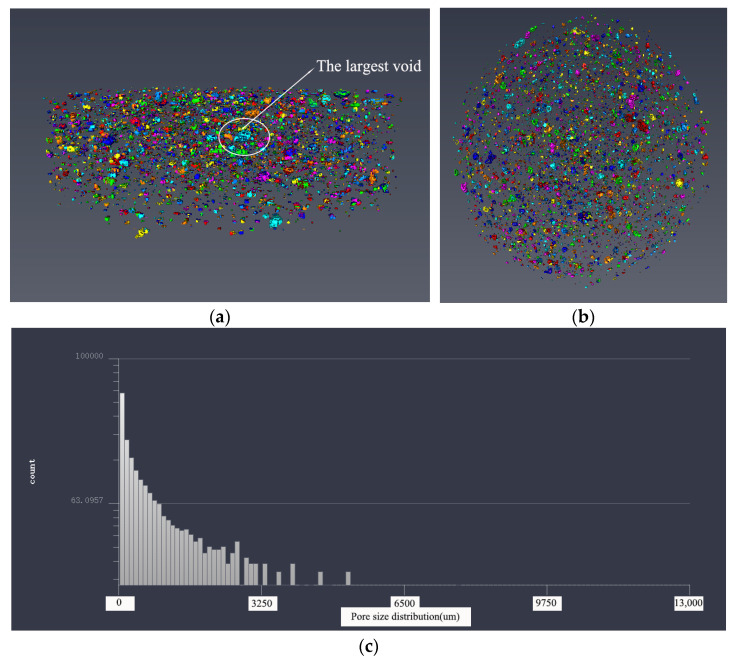
The 3D model of the pore structure of Group 2. (**a**) Side view; (**b**) vertical view; (**c**) pore distribution statistics.

**Figure 12:**
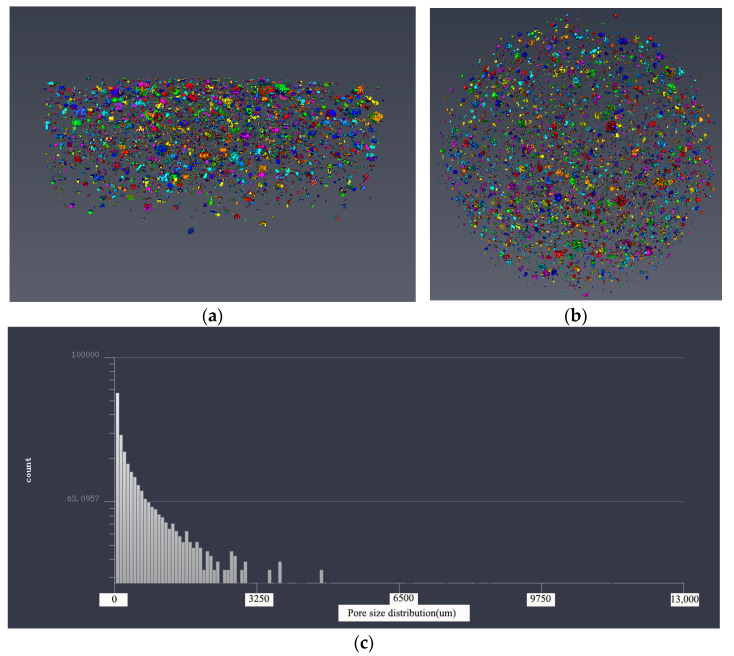
The 3D model of the pore structure of Group 3. (**a**) Side view; (**b**) vertical view; (**c**) pore distribution statistics.

**Figure 13:**
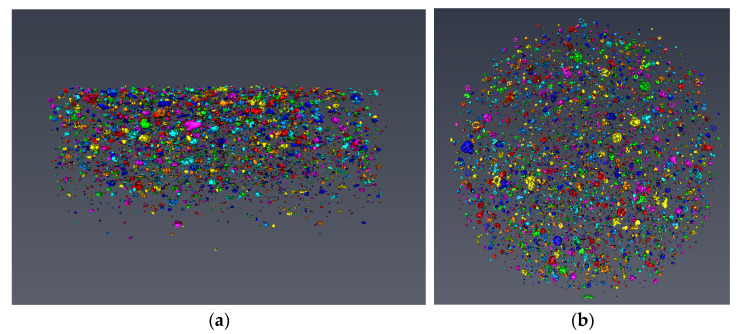

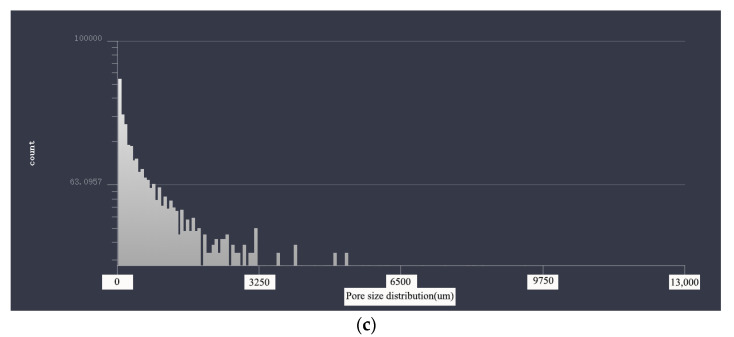
The 3D model of the pore structure of Group 4. (**a**) Side view; (**b**) vertical view; (**c**) pore distribution statistics.

**Figure 14:**
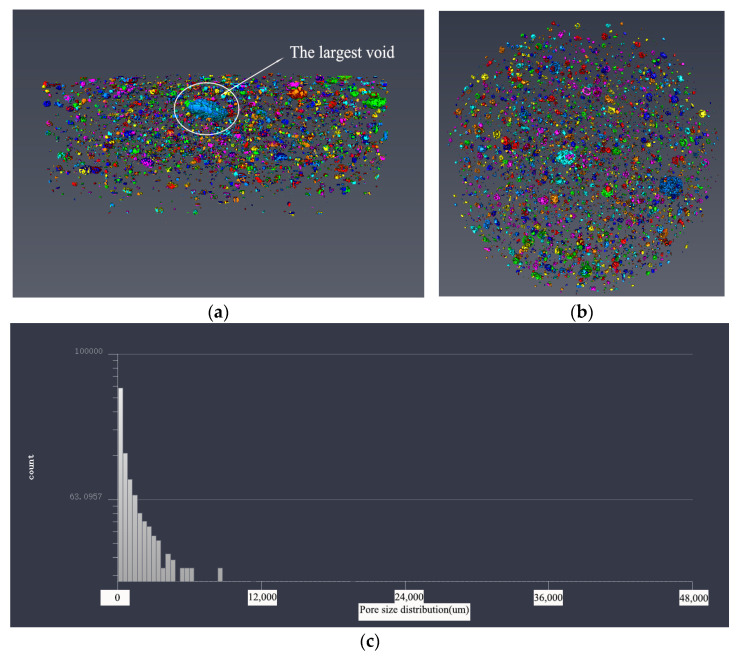
The 3D model of the pore structure of Group 5. (**a**) Side view; (**b**) vertical view; (**c**) pore distribution statistics.

**Figure 15:**
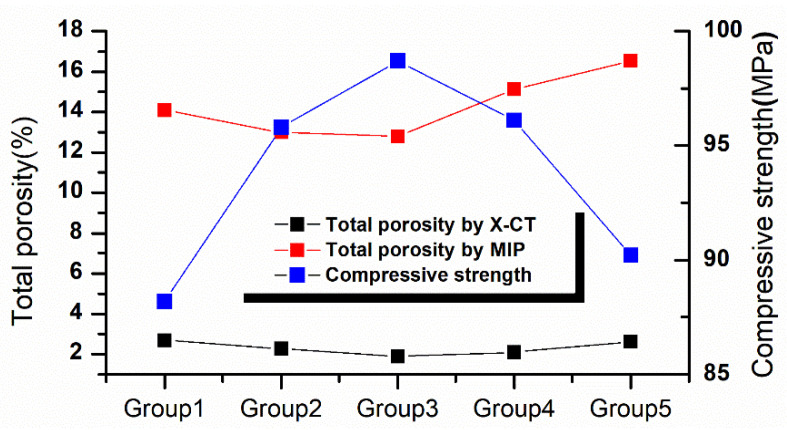
Comparative analysis of pore structure and mechanical properties.

In order to make a more intuitive analysis of the pore structure of five samples, the threshold adjustment and data segmentation were performed using Avizo software to obtain the 3D model of the pore structure. The pore structure 3D models of five samples are shown in Figure 11, Figure 12, Figure 13, Figure 14 and Figure 15. According to the pore structure 3D model characteristics, when the desert sand replacement increased from 0% to 50%, the total air voids content generally decreased. Overall, the content of larger air voids was also significantly reduced, and the number of smaller air voids increased. When desert sand replacement rate increased from 50% to 100%, the total air voids content generally increased. The largest air void in the five samples were clearly present in Group 5. The figure also shows that the upper end air voids of the sample were dense and mainly large size air voids, the lower ends of the sample were sparse and mainly small size air voids.

By measuring the size of the air voids in 3D model one can get the statistical data. The statistical results show that: The diameter size of Group 1 ranged from 254.273 to 4959.207 μm; the diameter size of Group 2 ranged from 176.27 to 4883.869 μm; the diameter size of Group 3 ranged from 233.89 to 3485.766 μm; the diameter size of Group 4 ranged from 176.27 to 3695.517 μm; the diameter size of Group 5 ranged from 176.27 to 8232.703 μm. It can be found that the minimum diameter of the air voids found in 3D model of the pore structure with the μX-CT technique is about 200 μm. The air voids in this pore size range are mainly more harmful pores in the pore classification, which are mainly caused by bubbles or insufficient hydration, and have great harmful effects on the strength of concrete. At the same density, the narrow pore size distribution is beneficial to increase the strength of concrete. This pore size distribution was obviously beneficial to improve the strength of Group 3.

The pore size distribution of five samples were derived from the pore structure 3D model characteristics, as shown in Figure 11c, Figure 12c, Figure 13c, Figure 14c and Figure 15c. It can be found that the number of air voids decreased with the increase of pore size. When the desert sand replacement rate was 0–75%, the number of air voids with pore size above 3250 μm decreased obviously, and the addition of desert sand reduced the number of large air voids and improved the pore structure. However, when the desert sand replacement rate was 100%, the number of larger air voids increased, and the maximum pore size was over 8000 μm.

Table 5 shows the pore structure statistic data calculated by Avizo software. The mean pore volume of the five samples showed a trend of decreasing before increasing, with a minimum of 0.1906 mm^3^ at a desert sand replacement rate of 50%. This means that the pore structure of fiber reinforced mortar after the addition of desert sand was somewhat optimized, the number of small air voids and micro air voids increased and large air voids were reduced. The minimum pore volume of the five samples was basically the same, and the maximum pore volume basically tended to decrease first and then increase. With the amount of desert sand incorporation, the volume of the sample maximum pore decreased significantly. With the increase of desert sand replacement rate, the maximum pore volume decreased obviously. When the desert sand replacement rate was 25%, 50%, and 75%, the maximum pore volume of concrete was 75.6%, 29.5%, and 17.9% of river sand concrete, respectively. However, when the desert sand replacement rate was 100%, there was a very large air void with a volume of 90.8732 mm^3^, which may be caused by uneven oscillation in the preparation process. This phenomenon was also shown in the 3D reconstruction model of pore structure of Group 5.

The pore structure characteristics obtained by 3D reconstruction model are basically the same as that calculated by 2D images. Obviously, it is an efficient and convenient method to analyze the pore structure of concrete by 3D modeling because of the huge amount of work needed to calculate the pore structure characteristics from 2D images. It can not only make the pore structure of samples become visualized and more intuitively observe the changes of the pore structure, but also quantitatively analyze the pore structure of samples through the data analysis method in the Avizo software.

### 3.4. Discussion

In the above discussion, the porosity of five samples was determined by MIP and μX-CT, respectively. The pore size measured by MIP was in the range of 1 nm–500 μm. With the increase of desert sand replacement rate, the porosity in this range first decreased and then increased. The pore structure was improved by the replacement of a certain amount of desert sand. The pore size measured by μX-CT was more than 200 μm, which is the more harmful pore in the pore size classification of concrete. The pore structure was improved by the replacement of a certain amount of desert sand. With the increase of desert sand replacement rate, the porosity in this range also tended to decrease first and then increase. Therefore, in the range of pore size from 1 nm to 8232 μm, with the increase of desert sand replacement rate, the pore size of fiber reinforced mortar showed a changing rule of increasing first and then decreasing. Therefore, MIP and μX-CT can be combined to systematically investigate the complete pore structure of fiber-reinforced mortar under different desert sand replacement rates.

Figure 15 shows the variation of compressive strength and porosity measured by MIP and μX-CT. With the increase of desert sand replacement rate, the compressive strength of concrete increases first and then decreases, which is contrary to the trend of porosity, indicating that the compressive strength of fiber reinforced mortar is inversely proportional to porosity. The decrease of porosity indicates that the content of pores in fiber reinforced mortar decreases, and the pore structure is optimized, thus improving the compressive strength of fiber reinforced mortar. When the replacement rate of desert sand is 100%, the compressive strength of fiber reinforced mortar is still higher than that of pure river sand, indicating that the pore structures of fiber reinforced mortar with 100% desert sand replacement rate are optimized compared with river sand fiber reinforced mortar. In this case, the porosity obtained by MIP is higher than that of river sand fiber reinforced mortar, and the porosity obtained by μX-CT is lower than that of river sand fiber reinforced mortar, indicating that the pore size above 200 μm has a greater impact on the mechanical properties of fiber reinforced mortar. When the replacement rate of desert sand is 50%, the pore structure of fiber reinforced mortar is the best and the compressive strength is the maximum.

## 4. Conclusions

In this study, the effect of desert sand on pore structure of fiber reinforced mortar were systematically investigated. The main conclusions are summarized as follows:

1. This paper studied the effect of desert sand on fiber reinforced mortar. The compressive strength increases first and then decreases at different rates with the increase of desert sand replacement rate. After curing for 28 days, relative to river sand mortar, the increment of compressive strength of fiber reinforced mortar with 25%, 50%, 75%, and 100% desert sand replacement rate is 8.61%, 11.90%, 8.96%, and 2.28%, respectively. Mortar with 50% desert sand replacement rate has the highest compressive strength, up to 98.7 MPa, which has a positive effect on the application of desert sand in concrete.
2. MIP and μX-CT techniques were used to study the pore structure of fiber reinforced mortar with different desert sand replacement rate. The changes of pore structure characteristics obtained by MIP and μX-CT are basically consistent. The MIP technique was used to study the pore structure characteristics from 1 nm to 500 μm, and the pore size changes are mainly distributed in the range of 1–100 nm and over 200 μm. It is found that when the replacement rate of desert sand exceeds 50%, the pore structure characteristics measured by MIP in the range of 1 nm to 100 nm are worse than those of the reference group, indicating that the pore structure characteristics are inferior to those of pure river sand fiber reinforced mortar. The μX-CT technique was used to investigate the pore structure characteristics above 200 μm. The pores in this range are more harmful pores and have a great influence on the strength of cement mortar. When the replacement rate of desert sand exceeds 50%, the pore structure becomes worse in the range of 200 μm, but it is still better than pure river sand group. This indicates that the pore over than 200 μm has a greater influence on the strength of fiber reinforced mortar.
3. Two methods were used to analyze the pore structure of samples based on μX-CT technique. The first method calculates the porosity of each CT image to obtain the porosity at different depths and total porosity. The second method models the pore structure based on Avizo software to obtain a three-dimensional model of the pore structure. The changes of pore structure characteristics obtained by the two methods are basically the same, and the porosity values remain the same. In comparison, it is an efficient and convenient method to analyze the pore structure of concrete by 3D modeling. It cannot only make the pore structure of samples become visualized and more intuitively observe the changes of the pore structure, but also quantitatively analyze the pore structure of samples through the data analysis method in the Avizo software.
4. The compressive strength of fiber reinforced mortar with different desert sand re-placement rate decreases with the increase of porosity. When the desert sand replacement rate is 50%, the sample has the lowest porosity, the best pore structure characteristics, and the highest compressive strength. The optimization of pore structure can effectively improve the microstructure of cement mortar, make the microstructure of cement mortar become more compact, and improve the compressive strength.

## Figures and Tables

**Table 1:** The chemical and physical properties of cement.

Items	Chemical Composition/%							Ignition Loss Rate	Specific Surface Area/(cm^2^/g)	Packing Density/(g/cm^3^)
	CaO	SiO_2_	Al_2_O_3_	MgO	Fe_2_O_3_	Na_2_O	SO_3_			
Cement	61.54	15.40	4.43	0.72	4.91	0.04	2.75	2.24	3500	3.1

**Table 2:** Mix design of fiber reinforced mortar with desert sand (mass ratio).

Group	Cement	Steel Fiber	Desert Sand Replacement Rate%	Desert Sand	River Sand	Deionized Water
1	1	0.30	0	\	1.2	0.45
2	1	0.30	25	0.3	0.9	0.45
3	1	0.30	50	0.6	0.6	0.45
4	1	0.30	75	0.9	0.3	0.45
5	1	0.30	100	1.2	\	0.45

**Table 3:** Porosity and cumulative pore volume of specimens of different mix proportions.

Group	1	2	3	4	5
Porosity (%)	14.0821	13.0013	12.7888	15.1259	16.5675
Cumulative pore volume (mL/g)	0.0729	0.0672	0.0662	0.0815	0.0928
Mean diameter (nm)	40,625	40,606	40,598	40,618	40,630

**Table 4:** Calculation results of porosity of different samples.

Start from the Top Surface	Porosity/%					Start from the Top Surface	Porosity/%				
	Group 1	Group 2	Group 3	Group 4	Group 5		Group 1	Group 2	Group 3	Group 4	Group 5
0–1 mm	3.41	3.04	2.72	2.85	3.15	25–26 mm	2.78	2.65	1.60	1.92	2.51
1–2 mm	3.34	3.37	2.53	2.70	3.12	26–27 mm	2.49	1.98	1.95	2.10	2.68
2–3 mm	3.58	3.34	2.69	2.74	3.45	27–28 mm	2.80	1.65	2.25	2.45	2.82
3–4 mm	3.62	3.17	2.70	3.19	3.41	28–29 mm	2.75	2.56	2.22	2.41	2.65
4–5 mm	3.88	3.26	2.61	3.21	3.29	29–30 mm	2.56	2.48	2.26	1.69	2.50
5–6 mm	3.56	3.11	2.70	3.20	3.13	30–31 mm	2.78	2.40	2.03	1.71	2.81
6–7 mm	3.45	2.97	2.66	3.33	3.27	31–32 mm	2.30	1.95	1.69	1.51	2.77
7–8 mm	3.41	2.99	2.91	2.89	2.91	32–33 mm	2.56	1.85	1.96	1.43	2.95
8–9 mm	3.51	2.96	2.69	3.04	3.79	33–34 mm	2.48	1.86	1.06	1.79	2.80
9–10 mm	3.55	2.84	3.21	2.74	3.96	34–35 mm	2.95	1.56	1.05	1.46	2.58
10–11 mm	3.50	2.74	2.63	2.83	4.00	35–36 mm	2.54	1.59	1.28	1.20	2.70
11–12 mm	3.56	2.79	2.65	2.98	3.74	36–37 mm	2.39	1.48	1.19	1.57	2.06
12–13 mm	3.34	2.99	2.74	2.90	3.27	37–38 mm	2.30	1.33	1.26	1.57	2.05
13–14 mm	3.59	3.12	2.50	2.96	2.97	38–39 mm	2.56	1.43	1.15	1.07	2.58
14–15 mm	3.45	3.05	2.65	3.02	2.90	39–40 mm	1.96	1.08	1.12	1.39	2.70
15–16 mm	3.56	2.65	2.25	1.92	2.53	40–41 mm	1.89	1.12	1.29	1.62	2.06
16–17 mm	3.55	2.75	2.36	1.71	2.49	41–42 mm	1.41	1.26	1.35	1.88	1.89
17–18 mm	3.23	2.99	2.71	2.57	2.42	42–43 mm	1.30	1.26	1.04	2.29	1.64
18–19 mm	3.26	3.01	1.60	2.58	3.14	43–44 mm	1.21	1.94	1.05	1.43	1.41
19–20 mm	3.10	2.91	1.51	1.79	3.05	44–45 mm	1.32	1.55	1.03	1.27	1.36
20–21 mm	2.77	2.90	1.79	1.60	2.99	45–46 mm	1.20	1.06	1.11	1.14	1.40
21–22 mm	2.78	2.81	1.65	1.92	2.60	46–47 mm	1.04	1.05	1.03	1.01	1.25
22–23 mm	2.51	2.86	1.95	2.33	2.53	47–48 mm	1.02	1.00	0.97	1.05	1.21
23–24 mm	2.65	2.65	2.26	1.79	2.42	48–49 mm	0.89	0.85	0.74	0.74	0.96
24–25 mm	2.61	2.61	1.89	1.60	2.73	49–50 mm	0.74	0.73	0.69	0.73	0.75
The upper area	3.51	3.02	2.68	2.91	3.30	\	\	\	\	\	\
The middle area	2.80	2.57	1.98	1.99	2.69	\	\	\	\	\	\
The bottom area	1.76	1.33	1.13	1.37	1.91	\	\	\	\	\	\
Average	2.70	2.27	1.89	2.09	2.64	\	\	\	\	\	\

**Table 5:** Pore structure characteristics modeling by Avizo software.

Group	1	2	3	4	5
**Porosity**	2.02%	1.92%	1.84%	1.89%	1.85%
**Pore size Distribution** **(** **μ** **m** **)**	254.273–4959.207	176.27–4883.869	233.89–3485.766	176.27–3695.517	176.27–8232.703
**Mean Pore Volume** **(** **mm^3^** **)**	0.2094	0.1960	0.1906	0.2009	0.2052
**Min Pore Volume** **(** **mm^3^** **)**	0.0412	0.0412	0.0412	0.0399	0.0401
**Max Pore Volume** **(** **mm^3^** **)**	87.8677	66.4778	25.9235	15.6972	90.8732

## Data Availability

The data underlying this article will be shared on reasonable request from the corresponding author.
